# Exploring the Pharmacological Mechanism of Duhuo Jisheng Decoction in Treating Osteoporosis Based on Network Pharmacology

**DOI:** 10.1155/2021/5510290

**Published:** 2021-04-05

**Authors:** Zhencheng Xiong, Can Zheng, Yanan Chang, Kuankuan Liu, Li Shu, Chi Zhang

**Affiliations:** ^1^Institute of Medical Technology, Peking University Health Science Center, Beijing, China; ^2^Peking University Third Hospital, Beijing, China; ^3^Biomedical Engineering Department, Peking University, Beijing, China; ^4^Central Laboratory, Peking University International Hospital, Beijing, China; ^5^Department of Orthopedics, Peking University International Hospital, Beijing, China; ^6^School of Chinese Materia Medica, Beijing University of Chinese Medicine, Beijing, China

## Abstract

**Objective:**

The purpose of this work is to study the mechanism of action of Duhuo Jisheng Decoction (DHJSD) in the treatment of osteoporosis based on the methods of bioinformatics and network pharmacology.

**Methods:**

In this study, the active compounds of each medicinal ingredient of DHJSD and their corresponding targets were obtained from TCMSP database. Osteoporosis was treated as search query in GeneCards, MalaCards, DisGeNET, Therapeutic Target Database (TTD), Comparative Toxicogenomics Database (CTD), and OMIM databases to obtain disease-related genes. The overlapping targets of DHJSD and osteoporosis were identified, and then GO and KEGG enrichment analysis were performed. Cytoscape was employed to construct DHJSD-compounds-target genes-osteoporosis network and protein-protein interaction (PPI) network. CytoHubba was utilized to select the hub genes. The activities of binding of hub genes and key components were confirmed by molecular docking.

**Results:**

174 active compounds and their 205 related potential targets were identified in DHJSD for the treatment of osteoporosis, including 10 hub genes (AKT1, ALB, IL6, MAPK3, VEGFA, JUN, CASP3, EGFR, MYC, and EGF). Pathway enrichment analysis of target proteins indicated that osteoclast differentiation, AGE-RAGE signaling pathway in diabetic complications, Wnt signaling pathway, MAPK signaling pathway, PI3K-Akt signaling pathway, JAK-STAT signaling pathway, calcium signaling pathway, and TNF signaling pathway were the specifically major pathways regulated by DHJSD against osteoporosis. Further verification based on molecular docking results showed that the small molecule compounds (Quercetin, Kaempferol, Beta-sitosterol, Beta-carotene, and Formononetin) contained in DHJSD generally have excellent binding affinity to the macromolecular target proteins encoded by the top 10 genes.

**Conclusion:**

This study reveals the characteristics of multi-component, multi-target, and multi-pathway of DHJSD against osteoporosis and provides novel insights for verifying the mechanism of DHJSD in the treatment of osteoporosis.

## 1. Introduction

Osteoporosis is characterized by low bone mass, impaired bone microstructure, increased bone fragility, and susceptibility to fracture and is also a systemic bone disease [1]. Osteoporosis can occur in different genders and ages, but it is more common in postmenopausal women and elderly men [2]. The serious consequences of osteoporosis are fragility fractures that can occur during minor trauma or daily activities, leading to an increase in disability and mortality [3]. Moreover, the treatment and nursing of osteoporosis and osteoporotic fracture need to invest huge manpower and material resources, and the cost is high, resulting in heavy family, social, and economic burden [4]. Therefore, early adoption of scientific prevention strategies and standardized treatment is very necessary. Among these, drug therapies such as bisphosphonates and denosumab are commonly used to treat osteoporosis by inhibiting the development, formation, and survival of osteoclasts [1]. Although these drugs are effective, high-dose or long-term use may cause serious adverse effects, such as gastrointestinal tolerance, atypical long bone fracture, jaw osteonecrosis, and acute renal failure [1]. Therefore, continuing to search for potential drugs with significant efficacy and high safety has become a consistent hot spot for the treatment of osteoporosis.

As we all know, traditional Chinese medicine (TCM) plays an important role in health maintenance in China and other Asian countries [5]. For a long time, TCM has been used as a complementary and alternative treatment option for patients with osteoporosis [6]. Systematic reviews and experimental studies have explored the efficacy and safety of TCM prescriptions in the treatment of osteoporosis, including Duhuo Jisheng Decoction (DHJSD), Xianling Gubao capsules, Liuwei Dihuang Decoction, and Erxian Decoction [2, 7–9].

DHJSD is a TCM recorded in Bei Ji Qian Jin Yao Fang of the Tang Dynasty for the treatment of “Bi Zheng,” usually consisting of the following 15 herbs, including Du Huo (*Radix Angelicae Pubescentis*), Sang Ji Sheng (*Herba Taxilli*), Qin Jiao (*Radix Gentianae Macrophyllae*), Fang Feng (*Radix Saposhnikoviae*), Xi Xin (*Herba Asari*), Fu Ling (*Poria Cocos*), Chuan Xiong (*Rhizoma Chuanxiong*), Bai Shao (*Radix Paeoniae Alba*), Du Zhong (*Cortex Eucommiae Ulmoidis*), Ren Shen (*Panax Ginseng*), Gan Cao (*Radix Glycyrrhizae*), Dang Gui (*Radix Angelicae Sinensis*), Niu Xi (*Radix Achyranthis Bidentatae*), Shu Di Huang (*Radix Rehmanniae Preparata*), and Rou Gui (*Cortex Cinnamomi*) [10]. Among them, Du Huo has the good effects of relieving the pain of “Bi Zheng,” dispelling cold and dehumidification, nourishing blood and Qi; Xi Xin, Fang Feng, and Qin Jiao dispel rheumatism, relax tendons and muscles, and benefit joints; Sang Ji Sheng, Du Zhong, Rou Gui, and Niu Xi nourish liver and kidney and strengthen bones and muscles; Dang Gui, Bai Shao, Shu Di Huang, and Chuan Xiong promote blood circulation; Ren Shen, Gan Cao, and Fu Ling strengthen spleen and supplement Qi [11]. The combination of the above herbs forms DHJSD, the classic bone injury prescription in TCM, it has the effect of nourishing the liver and kidney, benefiting Qi and blood, and stopping the pain of “Bi Zheng”, which obviously improves the microcirculation of the body [2]. According to the theory of Chinese medicine, “Bi Zheng” refers to the symptoms of numbness, soreness, and poor flexion of muscles and joints caused by external factors (wind, dampness, cold, heat) on the body surface, tendons, and veins [12]. Osteoporosis belongs to the category of “Gu Bi” and “Gu Lou” in TCM theory [13]. And the cause of osteoporosis is the deficiency of Qi and blood in the liver and kidney and the loss of nutrition in the tendons and bones, so the treatment should be to nourish the liver and kidney and strengthen the tendons and bones as the primary treatment [14]. Therefore, DHJSD has been widely used in China to treat rheumatoid arthritis, intervertebral disc disease, knee osteoarthritis, and osteoporosis, especially postmenopausal osteoporosis [2, 11, 14–17]. However, due to the ingredients contained, the mechanism of action of TCM is often elusive.

**Figure 1:**
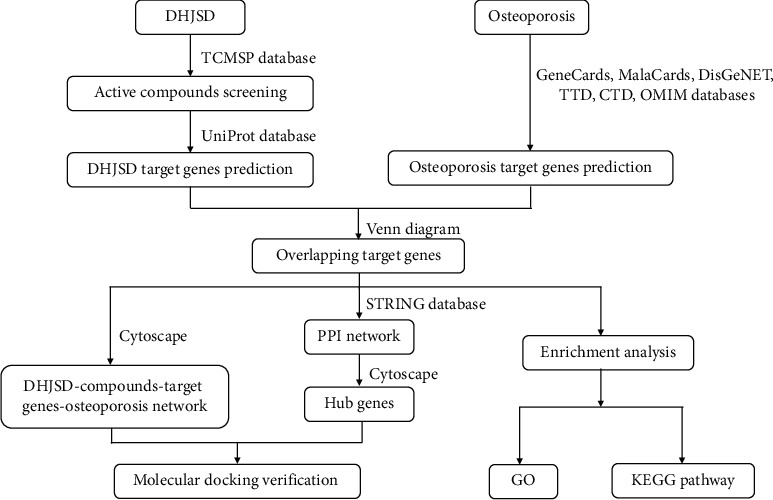
The schematic diagram of the present study to investigate potential mechanisms of DHJSD against osteoporosis.

The network pharmacology of TCM is a part of bioinformatics, and it is still a priori analysis method for studying the relationship between drugs, compounds, diseases, and targets [18]. Due to the characteristics of multi-component, multi-target, and multi-pathway of TCM, network pharmacology has been widely used to clarify the mechanism of action of TCM and provide researchers with new directions and strategies. In this work, we tried to use the network pharmacology method to reveal the active compounds of DHJSD, and the key genes and pathways of DHJSD against osteoporosis, which facilitated further research and development (Figure 1).

## 2. Materials and Methods

### 2.1. Screening of Potential Active Compounds and Related Targets in DHJSD

We used the Traditional Chinese Medicine Systems Pharmacology (TCMSP, Version: 2.3, https://tcmspw.com/tcmsp.php) database and the analysis platform [19], and input the names of 15 Chinese herbal medicines in DHJSD to obtain the corresponding compounds and related information. According to the absorption, distribution, metabolism, and excretion (ADME) protocols, the active compounds were screened, and the criteria were oral bioavailability (OB) ≥30 and drug-likeness (DL) ≥0.18 [20, 21]. Then, the potential target proteins of the selected active compounds were mined in TCSMP database to construct the potential target gene set of DHJSD. UniProt database (https://www.uniprot.org/) was used to obtain the unique corresponding gene names and UniProt ID of drug or disease-related targets [22]. The species was selected as “*Homo sapiens*.”

### 2.2. Mining of Osteoporosis-Related Targets

Osteoporosis-related targets were obtained through retrieving GeneCards (https://www.genecards.org/) [23], MalaCards (https://www.malacards.org/) [24], DisGeNet database (https://www.disgenet.org/, v7.0) [25], Therapeutic Target Database (TTD) (http://db.idrblab.net/ttd/) [26], Comparative Toxicogenomics Database (CTD) (http://ctdbase.org/, Last update by June, 2020) [27], and Online Mendelian Inheritance in Man (OMIM) (https://omim.org/, updated November 25, 2020) [28] using the keyword “osteoporosis.” GeneCards is a comprehensive, user-friendly database providing information on all annotated and predicted human genes, and we screen out targets with the relevance score ≥10 [23]. DisGeNET is a discovery platform that contains one of the largest public collections of genes and variants related to human diseases; targets with the score ≥0.1 were screened [25]. CTD is a powerful public database designed to improve people's understanding of how environmental exposure affects human health; targets with the inference score ≥20 were screened [27]. Finally, the potential targets obtained from the six databases mentioned above were integrated and de-duplicated to construct a set of targets relevant to osteoporosis.

### 2.3. Network Construction and Analysis

DHJSD-related targets and osteoporosis-related targets were entered separately into the Venn online tool (http://www.bioinformatics.com.cn/) for common targets, which were candidate targets of DHJSD against osteoporosis. The interaction between “DHJSD-active compounds-target genes-osteoporosis” was constructed by Cytoscape software (version 3.7.2) [29].

### 2.4. Protein-Protein Interaction (PPI) Analysis

PPI underlies most biological processes in living cells and is essential for understanding cell physiology in normal and disease states [10]. In this study, PPI network analysis of the obtained common targets was performed using the STRING database (http://string-db.org/; version 11) with the species limited to “*Homo sapiens*” and a confidence score >0.4 [30]. The PPI networks were constructed by Cytoscape software (version 3.7.2). Additionally, the plug-in 12 CytoHubba algorithms of Cytoscape software (Degree, Maximal Clique Centrality (MCC), Density of Maximum Neighborhood Component (DMNC), Maximum Neighborhood Component (MNC), Edge Percolated Component (EPC), Closeness, Betweenness, ClusteringCoefficient, EcCentricity, Radiality, Stress, BottleNeck) were used to select the first 10 nodes for finding the hub genes [31, 32].

### 2.5. Gene Ontology (GO) and Kyoto Encyclopedia of Genes and Genomes (KEGG) Pathway Enrichment Analysis

We performed GO and KEGG pathway enrichment analysis using the clusterProfiler package in *R* (R 4.0.2 for Windows) to identify the biological processes and signaling pathways involved in DHJSD in treating osteoporosis [33–35]. An adjusted *P* value of <0.05 was considered to identify the enriched terms.

### 2.6. Molecular Docking

The plug-in CytoHubba of Cytoscape software was used to screen top 10 hub genes [32]. In addition, Sankey diagram (http://sankeymatic.com/) was built with top 10 hub genes and relative active ingredients of DHJSD to find out key active ingredients. Sankey diagram discloses the relationship among herb, ingredients, and targets. Subsequently, molecular docking between top 10 hub genes and key active ingredients was carried out to predict the accuracy of the pivotal components and prediction targets using AutoDock Vina [36]. PubChem database (https://pubchem.ncbi.nlm.nih.gov/) and RCSB protein data (http://www.rcsb.org/) were selected to download the MOL2 format of ligands and PDB format of proteins [4]. Crystal of proteins were introduced to Pymol software (https://pymol.org/2/; version 2.4.1) to conduct dehydration and separation of ligands [37]. Subsequently, the crystal conducted was introduced to AutoDockTools to build a docking grid box of targets [1]. Molecular dockings were achieved via AutoDock Vina [37]. The lower Vina scores, one of the results of molecular docking, represent a more stable binding affinity of protein and ligand [38]. Eventually, the complexes of protein and compound were visualized by Pymol software.

## 3. Results

### 3.1. Screening of Active Compounds in DHJSD

A total of 1939 compounds in DHJSD were retrieved through the TCMSP database, of which 99 were from Du Huo, 46 were from Sang Ji Sheng, 27 were from Qin Jiao, 173 were from Fang Feng, 192 were from Xi Xin, 34 were from Fu Ling, 189 were from Chuan Xiong, 85 from Bai Shao, 147 from Du Zhong 190 from Ren Shen, 280 from Gan Cao, 125 from Dang Gui, 176 from Niu Xi, 76 from Shu Di Huang, and 100 from Rou Gui. According to the criteria of OB ≥30% and DL ≥0.18, a total of 240 (after removing duplication: 209) active compounds of DHJSD were screened, of which 9 were from Du Huo, 2 were from Sang Ji Sheng, 2 were from Qin Jiao, 18 were from Fang Feng, and 8 were from Xi Xin, 15 from Fu Ling, 7 from Chuan Xiong, 13 from Bai Shao, 28 from Du Zhong, 22 from Ren Shen, 92 from Gan Cao, 2 from Dang Gui, 20 from Niu Xi, and 2 from Shu Di Huang. However, there is no compound in Rou Gui that meets the screening criteria.  Table 1 shows the basic information of some active compounds in DHJSD.

### 3.2. Mining of the Putative Target Genes for DHJSD

By using the compound-target search function of TCMSP database, it was confirmed that 179 of the 209 active compounds in DHJSD possess target proteins. Subsequently, UniProt database was used to convert the target protein predicted by the biologically active compound of DHJSD into gene name. Finally, 267 putative target genes were identified.

### 3.3. Mining of the Potential Therapeutic Targets of DHJSD in Treating Osteoporosis

**Figure 2:**
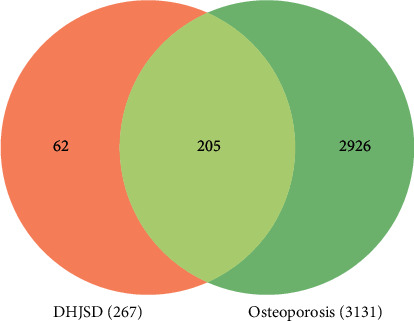
Venn diagram of DHJSD-related targets and osteoporosis-related targets.

**Figure 3:**
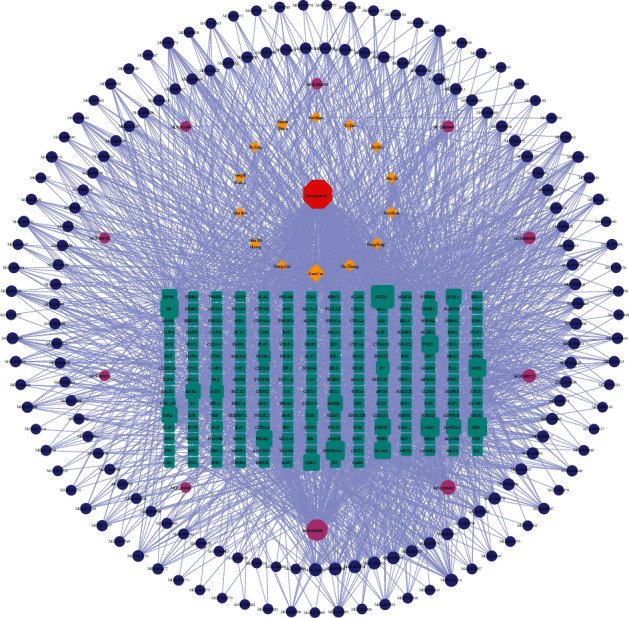
“DHJSD-active compounds-target genes-osteoporosis” network. The red octagon represents the disease; the brown diamond represents the herbs contained in DHJSD; the cyan rectangle represents the potential targets; the blue ellipse belongs to the herbs contained in DHJSD; and the purple ones are common ingredients. The line between two nodes represents the interaction; the size of each node represents the number of connections.

A total of 3131 potential therapeutic targets of osteoporosis were obtained by searching GeneCards, MalaCards, DisGeNET, TTD, CTD, and OMIM databases. The Venn diagram was drawn by using Venn online platform, which is derived from the targets regulated by the active ingredients of DHJSD and the potential targets of osteoporosis. Subsequently, a total of 205 core targets were obtained, which were the potential therapeutic targets of DHJSD in the treatment of osteoporosis, as shown in Figure 2. The “DHJSD-active compounds-target genes-osteoporosis” network of DHJSD against osteoporosis was constructed by Cytoscape software (Figure 3). In this case, the sub-network of “active compounds-target genes” includes 379 nodes and 1856 edges. We ranked the target genes according to the number of targeting nodes.  Table 2 shows the basic information of some key targets of DHJSD against osteoporosis.

### 3.4. Construction of the PPI Network and Mining of the Hub Genes of DHJSD in Treating Osteoporosis

**Figure 4:**
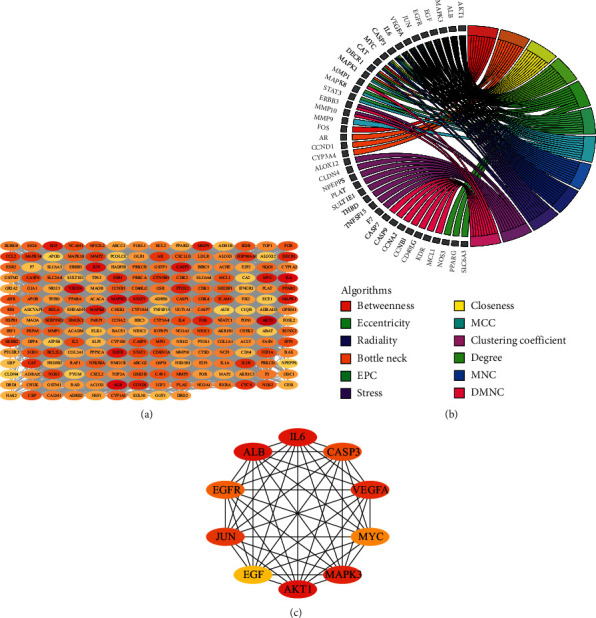
The PPI network of potential targets of DHJSD in the treatment of osteoporosis. (a) The PPI network from STRING was further analyzed using Cytoscape software (the line between two nodes indicates the interaction. The darker the color of the node, the better the relationship between them). (b) Chord diagram of the corresponding relationship between the top 10 genes and 12 CytoHubba algorithms. (c) The top 10 hub genes were identified by Degree (the darker the color of the node, the greater the degree).

**Figure 5:**
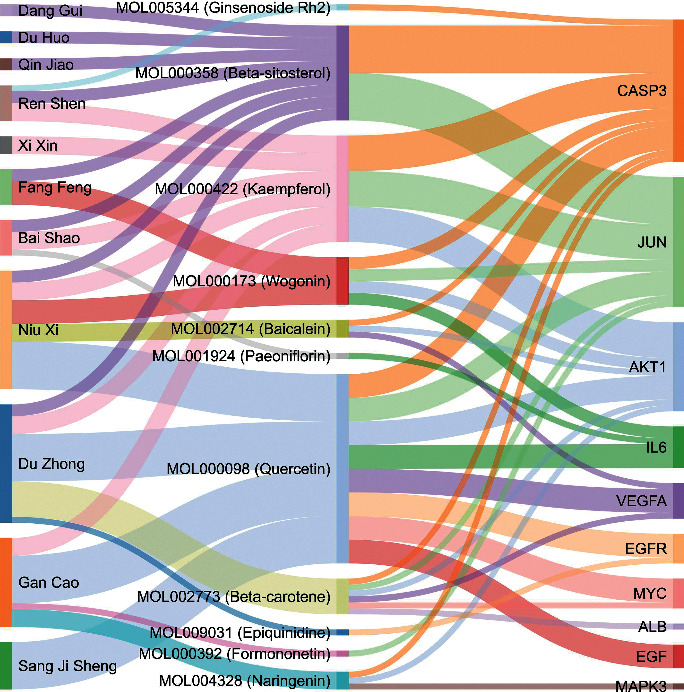
Sankey diagram. The colored vertical bars on the left represent the different herbs contained in DHJSD, the middle bars represent the active ingredients of the herbs, and the right bars represent the target genes. The size of the bars and the thickness of the lines represent the number of interactions.

A total of 205 potential target genes of DHJSD in the treatment of osteoporosis were input into STRING database to obtain PPI network. The network involves 205 nodes and 4078 edges. Then, the obtained data was imported into Cytoscape 3.7.2 version for further visualization (Figure 4(a)). Subsequently, according to the 12 CytoHubba algorithms of Cytoscape software, including Degree, MCC, DMNC, MNC, EPC, Closeness, Betweenness, ClusteringCoefficient, EcCentricity, Radiality, Stress, and BottleNeck, the top 10 hub genes of DHJSD for treating osteoporosis were selected based on the above results. The top 10 genes calculated based on 12 algorithms contain a total of 41 different genes. We showed the relationship between these genes and the corresponding algorithms in the form of Chord diagrams (Figure 4(b)). Then, we sorted the genes according to the number of algorithms corresponding to them and got the top 10 hub genes, and the results were consistent with the results of the Degree algorithm (Figure 4(c)). The top 10 hub genes contained AKT1, ALB, IL6, MAPK3, VEGFA, JUN, CASP3, EGFR, MYC, and EGF. In addition, the Sankey diagram was constructed using the top 10 hub genes and the relative active compounds of DHJSD, of which MOL000098 (Quercetin) targets most hub genes (Figure 5).

### 3.5. GO and KEGG Pathway Enrichment Analysis

**Figure 6:**
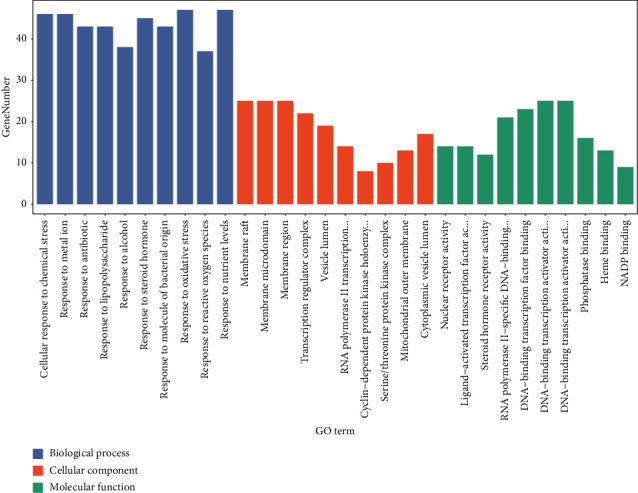
Barplot of the top 10 GO enrichment items. The GO enrichment items (BP, CC, MF) are arranged from left to right according to the adjusted *P* value.

In order to elucidate the biological mechanisms of DHJSD against osteoporosis, GO and KEGG pathway enrichment analysis was performed by using clusterProfiler in R. The 205 potential targets of DHJSD for treating osteoporosis were input into the *R*, and a total of 2590 GO terms (adjusted, *P* < 0.05) were obtained, including 2320 biological process (BP) terms and 179 molecular function (MF) terms and 91 cellular component (CC) terms. Based on the adjusted *P* value from small to large, the top 10 GO-BP terms were mainly enriched in cellular response to chemical stress (GO:0062197), response to metal ion (GO:0010038), response to antibiotic (GO:0046677), response to lipopolysaccharide (GO:0032496), response to alcohol (GO:0097305), response to steroid hormone (GO:0048545), response to molecule of bacterial origin (GO:0002237), response to oxidative stress (GO:0006979), response to reactive oxygen species (GO:0000302), and response to nutrient levels (GO:0031667). The top 10 GO-CC terms were mainly enriched in membrane raft (GO:0045121), membrane microdomain (GO:0098857), membrane region (GO:0098589), transcription regulator complex (GO:0005667), vesicle lumen (GO:0031983), RNA polymerase II transcription regulator complex (GO:0090575), cyclin-dependent protein kinase holoenzyme complex (GO:0000307), serine/threonine protein kinase complex (GO:1902554), mitochondrial outer membrane (GO:0005741), and cytoplasmic vesicle lumen (GO:0060205). The top 10 GO-MF terms were mainly enriched in nuclear receptor activity (GO:0004879), ligand-activated transcription factor activity (GO:0098531), steroid hormone receptor activity (GO:0003707), RNA polymerase II-specific DNA-binding transcription factor binding (GO:0061629), DNA-binding transcription factor binding (GO:0140297), “DNA-binding transcription activator activity, RNA polymerase II-specific” (GO:0001228), DNA-binding transcription activator activity (GO:0001216), phosphatase binding (GO:0019902), heme binding (GO:0020037), and NADP binding (GO:0050661). Finally, the top 10 GO enrichment terms were selected and visualized using bar diagram, as shown in Figure 6.  Table 3 shows the top 10 GO enrichment items.

**Figure 7:**
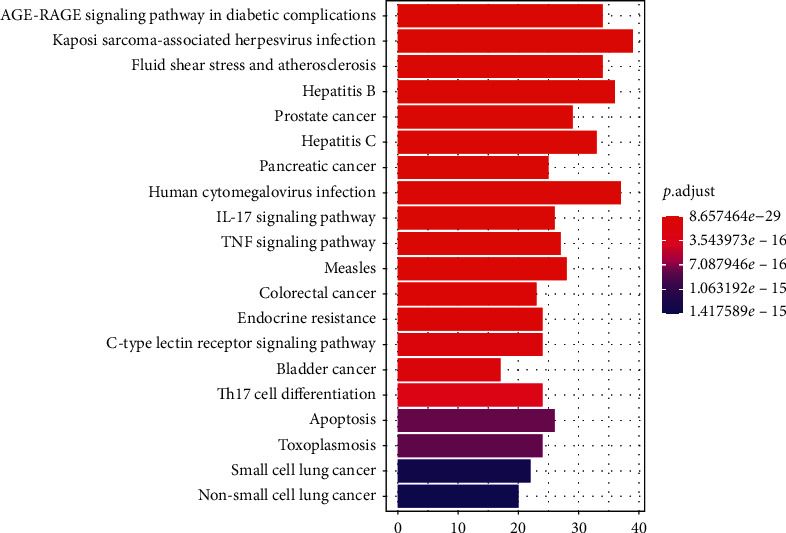
Barplot of the top 20 KEGG pathway enrichment items. The color of each bar represents the adjusted *P* value, and the length represents the number of enriched genes.

**Figure 8:**
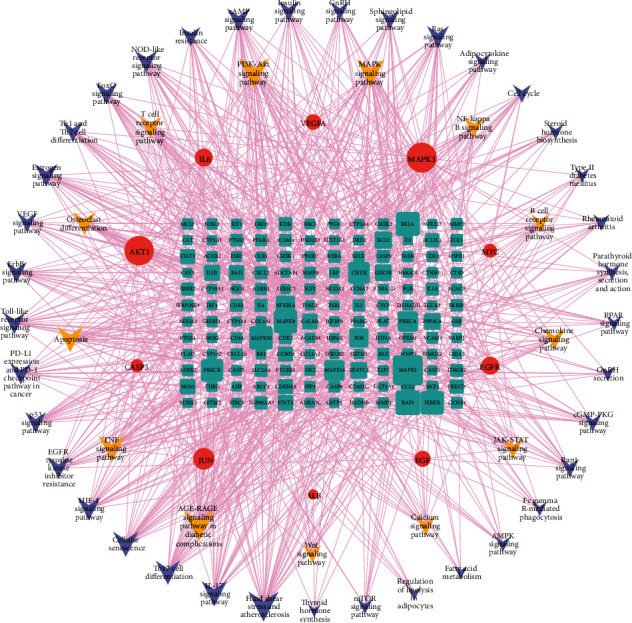
“Target genes-pathways” network. The cyan ellipse represents the potential targets of DHJSD against osteoporosis; the red ellipse represents the top 10 hub genes of DHJSD against osteoporosis; the brown arrow represents potential osteoporosis-related pathways; the light blue arrow represents osteoporosis-related pathways. The line between two nodes represents the interaction; the size of each node represents the number of connections. The lines consistent with the color of each hub gene represent the pathways enriched by that gene.

Additionally, a total of 170 enriched KEGG pathways (adjusted, *P* < 0.05) were obtained, the top 10 including AGE-RAGE signaling pathway in diabetic complications (hsa04933), Kaposi sarcoma-associated herpesvirus infection (hsa05167), fluid shear stress and atherosclerosis (hsa05418), hepatitis B (hsa05161), prostate cancer (hsa05215), hepatitis C (hsa05160), pancreatic cancer (hsa05212), human cytomegalovirus infection (hsa05163), IL-17 signaling pathway (hsa04657), and TNF signaling pathway (hsa04668). The top 20 KEGG pathway enrichment terms were selected and visualized using bar diagram, as shown in Figure 7. Next, we searched the KEGG pathway database for osteoporosis, looking for potential pathways related to osteoporosis in the enriched 170 pathways. A total of 50 pathways were selected that may be associated with osteoporosis, and then we proceeded to build a network map of target genes and pathways using Cytoscape software (Figure 8). These include 13 of the pathways most strongly associated with osteoporosis, including osteoclast differentiation (hsa04380), AGE-RAGE signaling pathway in diabetic complications (hsa04933), Wnt signaling pathway (hsa04310), MAPK signaling pathway (hsa04010), apoptosis (hsa04210), chemokine signaling pathway (hsa04062), T cell receptor signaling pathway (hsa04660), B cell receptor signaling pathway (hsa04662), PI3K-Akt signaling pathway (hsa04151), JAK-STAT signaling pathway (hsa04630), calcium signaling pathway (hsa04020), NF-kappa B signaling pathway (hsa04064), and TNF signaling pathway (hsa04668).  Table 4 shows the enriched 50 possible related pathways of osteoporosis.

### 3.6. Molecular Docking Results

**Figure 9:**
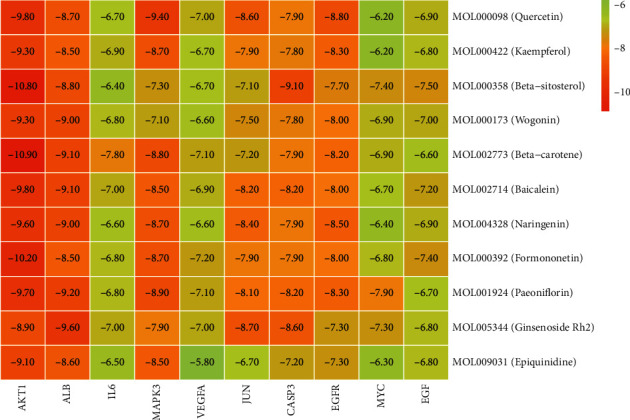
Heatmap of the docking scores of the active compounds of DHJSD and the target proteins.

**Figure 10:**
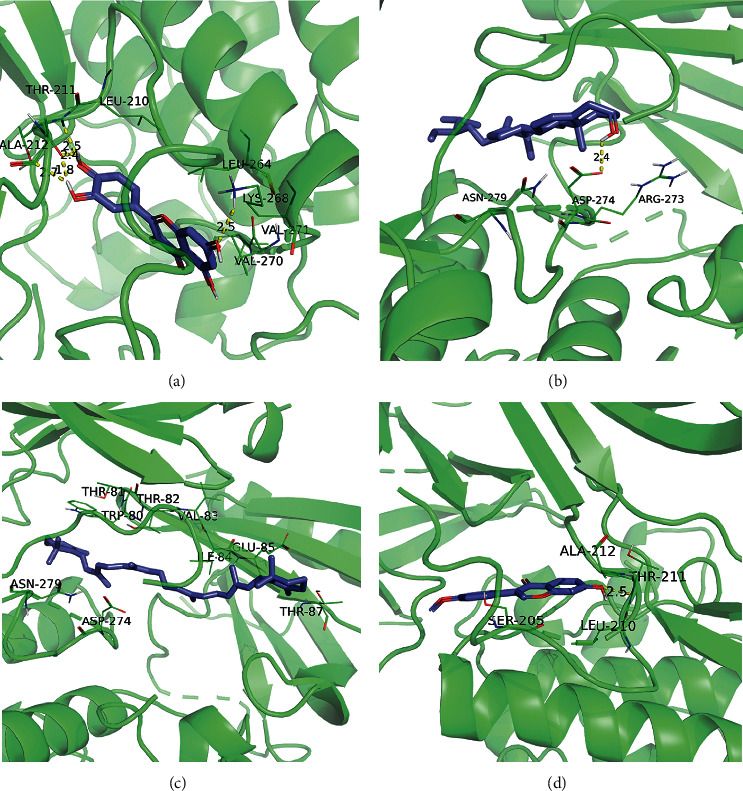
Molecular docking results of “bioactive compound-hub gene.” (a) Quercetin to AKT1; (b) Beta-sitosterol to AKT1; (c) Beta-carotene to AKT1; (d) Formononetin to AKT1.

According to the results of the Sankey diagram, we carried out molecular docking of the core target protein and active compound involved. Molecular docking between top 10 target proteins (AKT1, ALB, IL6, MAPK3, VEGFA, JUN, CASP3, EGFR, MYC, EGF) and key active compounds (Quercetin, Kaempferol, Beta-sitosterol, Wogonin, Beta-carotene, Baicalein, Naringenin, Formononetin, Paeoniflorin, Ginsenoside Rh2, and Epiquinidine) was carried out using AutoDock Vina. The docking scores of the strongest affinity of 10 core target proteins and 11 key active compounds were visualized using the heatmap, as shown in Figure 9. The binding energy between target proteins and the active compounds was approximately between −5.8 and −10.9 kcal mol^−1^. AKT1, ALB, MAPK3, JUN, CASP3, and EGFR have stronger docking energy. The remaining target proteins also have relatively strong docking energy, which means that the compounds in DHJSD bind well to 10 core target proteins. Eventually, we chose the top 4 target protein macromolecules and small compound molecules with the best docking affinity for visualization with Pymol (Figure 10).

## 4. Discussion

Osteoporosis is a degenerative disease, which increases with age. With the prolongation of human life and the coming of aging society, osteoporosis has become an important health problem [1]. Considering the threat of the aging of the global population and the increasing side effects of clinical drugs, finding potential drugs for the prevention and treatment of osteoporosis from natural products is a novel treatment strategy [2]. DHJSD is an extremely common TCM that has been used in China to treat patients with osteoporosis, especially postmenopausal osteoporosis [2]. However, the mechanism of action of TCM is usually elusive due to the ingredients it contains. It is of great significance to explore the molecular mechanism of TCM by using systematic and normative bioinformatics methods to mine multiple databases to integrate and analyze the information of target proteins and compounds [4]. A systematic network pharmacological method for determining molecular biological networks has been developed, which may be used to discover new therapeutic effects of drugs derived from medicinal plants. In this study, based on systematic network pharmacology, including ADME system assessment, PPI network analysis, GO and KEGG pathway analysis, and molecular docking verification, the active ingredients and potential targets of DHJSD in the treatment of osteoporosis were evaluated.

According to the principle of ADME (setting OB ≥30 and DL ≥0.18) [20], we searched the TCMSP database and screened a total of 209 active compounds (after deduplication) from the 15 herbs contained in DHJSD (Rou Gui did not find a compound that meets the requirements). By using the compound-target search function of the TCMSP database, 267 target proteins were finally found from 209 active compounds (30 of which did not find the corresponding target protein). Then, we transformed the full name of the target protein into the gene ID through UniProt database. Through searching 6 disease*-*target databases (GeneCards, MalaCards, DisGeNET, TTD, CTD, and OMIM), a total of 3131 potential osteoporosis treatment targets were obtained. Finally, we obtained 205 potential target genes of DHJSD in the treatment of osteoporosis. The network of DHJSD against osteoporosis was built, which was involved in 394 nodes and 2265 interactions. Furthermore, PPI network has been constructed involved in 205 nodes and 4078 edges. Top 10 hub genes were revealed by weighing the CytoHubba 12 algorithms of Cytoscape software (Degree, MCC, DMNC, MNC, EPC, Closeness, Betweenness, ClusteringCoefficient, EcCentricity, Radiality, Stress, and BottleNeck), including AKT1, ALB, IL6, MAPK3, VEGFA, JUN, CASP3, EGFR, MYC, and EGF.

AKT1 is one of three closely related serine/threonine protein kinases (AKT1, AKT2, and AKT3), which regulates many physiological processes including metabolism, proliferation, cell survival, and angiogenesis [39]. A study published in 2012 showed that AKT1 may be a regulator of the differentiation and function of osteoblasts and osteoclasts [40]. IL6 is an important inflammatory factor, and its function involves a variety of inflammation-related disease states, including diabetes mellitus susceptibility and systemic juvenile rheumatoid arthritis [41]. A study published in 2018 showed that the upregulation of IL6 expression is an important factor in promoting the osteogenic differentiation of adipose-derived stem cells [42]. ALB is the most important protein in human plasma, which maintains the body's nutrition and osmotic pressure, and acts as a carrier protein for a variety of endogenous molecules (including hormones, fatty acids, and metabolites) and exogenous drugs [43]. A retrospective study suggested that preoperative ALB levels may help predict complications after osteoporotic vertebral compression fractures [43]. MAPK3 is a member of the MAP kinase family and plays a role in the signal cascade that responds to various extracellular signals to regulate various cellular processes, such as proliferation, differentiation, and cell cycle progression [44]. Studies have shown that MAPK3 promotes the expression of RUNX2, and targeting MAPK3 can affect osteoblast differentiation [44]. VEGFA is a member of the PDGF/VEGF growth factor family, which is active in angiogenesis, angiogenesis, and endothelial cell growth, and induces endothelial cell proliferation, promotes cell migration, and inhibits cell apoptosis [45]. By inhibiting the expression of VEGFA, miR-16-5p could exert an anti-osteogenesis effect [45]. The human JUN gene encodes a protein that is highly similar to the viral protein, which directly interacts with specific target DNA sequences to regulate gene expression [46]. By activating the c-Fos/c-Jun pathway, IL-7/IL-7R could promote RANKL-mediated osteoclast formation and bone resorption and induce bone loss in ovariectomized mice [46]. The protein encoded by the CASP3 gene is a cysteine-aspartic acid protease, which plays a vital role in the execution-phase of cell apoptosis [47]. By targeting CASP3 and activating the PI3K-Akt signaling pathway, the overexpression of miR-378 could attenuate the osteogenic differentiation inhibited by high glucose [47]. The protein encoded by the EGF gene is a member of the epidermal growth factor superfamily, which acts as an effective mitogenic factor and plays an important role in the growth, proliferation, and differentiation of various cell types [48]. EGFR gene encodes a protein that is a receptor for members of the epidermal growth factor family [48]. Studies have shown that inhibition of the EGFR signaling pathway inhibits the expression of the enhancer of zeste homolog 2 (Ezh2) through the ERK1/2 pathway, thereby promoting the senescence of osteoprogenitor cells [48]. MYC is a proto-oncogene and encodes a nuclear phosphoprotein, which plays a role in cell cycle progression, apoptosis, and cell transformation [49]. In a mouse model of osteoporosis, both the loss of MYC and the pharmacological inhibitory effect of ERR*α* reduced bone loss [49]. The main physiological processes regulated by proteins encoded by the top 10 target genes include inflammatory response, immune response, cell proliferation, differentiation, apoptosis, migration, cell cycle progression, endocrine metabolism, angiogenesis, growth, and nutrition. Based on the above analysis results, it is also speculated that DHJSD against osteoporosis may play a role through the above process.

The one-to-one correspondence between the top 10 hub genes and the corresponding active compounds contained in DHJSD had been shown using the Sankey diagram. The relative active compounds include MOL000098 (Quercetin), MOL000422 (Kaempferol), MOL000358 (Beta-sitosterol), MOL000173 (Wogonin), MOL002773 (Beta-carotene), MOL002714 (Baicalein), MOL004328 (Naringenin), MOL000392 (Formononetin), MOL001924 (Paeoniflorin), MOL005344 (Ginsenoside Rh2), and MOL009031 (Epiquinidine). Among them, Quercetin targets most central genes. Quercetin is a member of the flavonoid family isolated from onion, apple, grape, tea, and many kinds of Chinese herbal medicines, which seems to have obvious anti-osteoporosis properties [50]. Studies have shown that Quercetin alone or in combination with alendronate could prevent glucocorticoid-induced osteoporosis through its bone formation stimulation [50]. Quercetin promotes bone marrow mesenchymal stem cells (BMSCs) proliferation and osteogenic differentiation, improves the in vitro model of osteoporosis, and provides protection against TNF-*α*-induced impairment of BMSC osteogenic function [51].

Then, we performed the GO and KEGG pathway enrichment analysis of genes that DHJSD against osteoporosis. Based on the adjusted *P* value from small to large, the top 3 GO-BP terms were mainly enriched in cellular response to chemical stress, response to metal ion, and response to antibiotic. The top 3 GO-CC terms were mainly enriched in membrane raft, membrane microdomain, and membrane region. The top 3 GO-MF terms were mainly enriched in nuclear receptor activity, ligand-activated transcription factor activity, and steroid hormone receptor activity. Based on the adjusted *P* value from small to large, the top 10 KEGG pathways were mainly enriched in AGE-RAGE signaling pathway in diabetic complications, Kaposi sarcoma-associated herpesvirus infection, fluid shear stress, and atherosclerosis, hepatitis B, prostate cancer, hepatitis C, pancreatic cancer, human cytomegalovirus infection, and IL-17 signaling pathway. We searched for osteoporosis in the KEGG pathway database and found that there are ten pathways directly related to osteoporosis and related diseases, including osteoclast differentiation (hsa04380), AGE-RAGE signaling pathway in diabetic complications (hsa04933), Wnt signaling pathway (hsa04310), MAPK signaling pathway (hsa04010), apoptosis (hsa04210), chemokine signaling pathway (hsa04062), T cell receptor signaling pathway (hsa04660), B cell receptor signaling pathway (hsa04662), endocrine and other factor-regulated calcium reabsorption (hsa04961), and mineral absorption (hsa04978). The first eight pathways are in the list of KEGG pathway enrichment analysis we have done. Six of the top 10 hub genes (AKT1, MAPK3, JUN, CASP3, IL6, and VEGFA) are enriched in AGE-AGE signaling pathway in diabetic complications, three of the top 10 hub genes (AKT1, MAPK3, and JUN) are enriched in osteoclast differentiation, eight of the top 10 hub genes (AKT1, MAPK3, VEGFA, JUN, CASP3, EGFR, MYC, and EGF) are enriched in MAPK signaling pathway, two of the top 10 hub genes (JUN, MYC) are enriched in Wnt signaling pathway, four of the top 10 hub genes (AKT1, MAPK3, JUN, and CASP3) are enriched in Apoptosis, two of the top 10 hub genes (AKT1, MAPK3) are enriched in Chemokine signaling pathway, and three of the top 10 hub genes (AKT1, MAPK3, and JUN) are enriched in T cell receptor signaling pathway and B cell receptor signaling pathway.

In addition, the enriched pathways related to the above pathways in the KEGG database include PI3K-Akt signaling pathway (hsa04151; enriched genes: AKT1, IL6, MAPK3, VEGFA, EGFR, MYC, EGF), JAK-STAT signaling pathway (hsa04630; enriched genes: AKT1, IL6, EGFR, MYC, EGF), calcium signaling pathway (hsa04020; enriched genes: EGFR, EGF), TNF signaling pathway (hsa04668; enriched genes: AKT1, IL6, MAPK3, JUN, and CASP3), and NF-kappa B signaling pathway (hsa04064). Li et al. [52] found that, through the PI3K-Akt signaling pathway, the knockdown of LNC_000052 could promote BMSCs osteogenesis, proliferation, and migration and inhibit cell apoptosis. Studies have shown that miR-10b could promote osteogenic differentiation and increase bone formation through the TGF-*β* signaling pathway [53]. In addition, JAK-STAT signaling pathway plays a role in the differentiation of osteoblasts and osteoclasts [54]. Jin et al. [55] found that sclareol prevents bone loss caused by ovariectomy in vivo by inhibiting NF-*κ*B and MAPK/ERK signaling pathways and inhibits osteoclast production in vitro. Zha et al. [56] found that miR-920 targets HOXA7 through the MAPK signaling pathway to promote the osteogenic differentiation of human bone mesenchymal stem cells. It is well known that there are many pathways that have a large or small relationship with osteoporosis, and these pathways are classified mainly as bone metabolism, inflammatory response, immune response, endocrine system, and cell apoptosis [57].

By mining the database, we obtained the compounds contained in each herb in DHJSD, and then we predicted the potential targets of DHJSD by these compounds, and then we performed KEGG pathway enrichment analysis to obtain the potential pathways of action of DHJSD based on these targets. Therefore, the above obtained targets and signaling pathways should be relevant to the functions of the herbs in DHJSD. It has been shown that the therapeutic effect of the herb Du Huo in osteoporotic rats is associated with the activation of Wnt/*β*-catenin signaling pathway to promote bone formation [58]. Qin Jiao extract showed a better inhibitory effect on adjuvant arthritis rats, which may be related to the inhibition of JAK2/STAT3 signaling pathway [59]. Fu Ling significantly ameliorated renal injury in db/db mice, and the mechanism may be related to the inhibition of p38 MAPK phosphorylation and the activation of PPAR*γ* pathway [60]. These results are consistent with the functional annotations of Du Huo (strengthening bones and reducing back and knee pain), Qin Jiao (dispelling rheumatism), and Fu Ling (strengthening the spleen and benefiting the kidney) in TCM theory. The number of studies on the targets and pathways of other herbs for the treatment of osteoporosis is currently very limited, and this becomes our next research direction. By using modern pharmacological methods to explore the specific mechanisms by which DHJSD exerts its therapeutic effects, it is more beneficial to understand and complement TCM theories, and thus to uncover the essence of TCM and find alternative therapies for diseases.

## 5. Conclusion

By using the network pharmacology approach, we have studied the potential targets of DHJSD and the underlying mechanism of its anti-osteoporosis effect, which has the characteristics of multi-component, multi-target, and multi-pathway. AKT1, ALB, IL6, MAPK3, VEGFA, JUN, CASP3, EGFR, MYC, and EGF may be potential targets of DHJSD in treating osteoporosis. According to the results of enrichment analysis of KEGG pathway, we found pathways closely related to the pathological process of osteoporosis, mainly including AGE-RAGE signaling pathway in diabetic complications, osteoclast differentiation, MAPK signaling pathway, Wnt signaling pathway, PI3K-Akt signaling pathway, JAK-STAT signaling pathway, and TNF signaling pathway. Therefore, this study reveals that the anti-osteoporosis effect of DHJSD may be based on its direct or indirect regulation of the above-mentioned potential targets and pathways. DHJSD may provide a promising direction for future research, so enough relevant experimental research verification is needed, which is very important for revealing its exact regulatory mechanism.

## Figures and Tables

**Table 1:** Basic information of some active compounds in DHJSD.

Molecule ID	Molecule name	OB (%)	DL	2D structure	Source	PubChem CID
MOL001924	Paeoniflorin	53.87	0.79	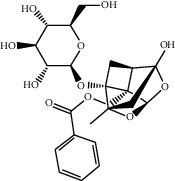	Bai Shao (*Radix Paeoniae Alba*)	442534
MOL000492	Cianidanol	54.83	0.24	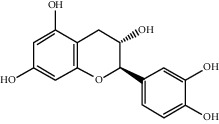	Bai Shao	9064
MOL002135	Myricanone	40.60	0.51	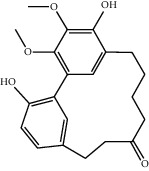	Chuan Xiong (*Rhizoma Chuanxiong*)	161748
MOL001494	Ethyl linoleate	42.00	0.19	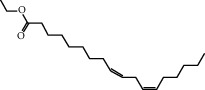	Chuan Xiong	5282184
MOL003608	O-acetylcolumbianetin	60.04	0.26	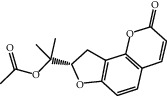	Du Huo (*Radix Angelicae Pubescentis*)	161409
MOL004780	Angelicone	30.99	0.19	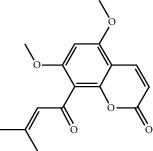	Du Huo	616303
MOL002773	Beta-carotene	37.18	0.58	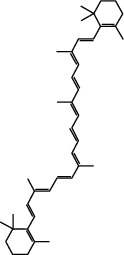	Du Zhong (*Cortex Eucommiae Ulmoidis*)	5280489
MOL009031	Epiquinidine	68.22	0.40	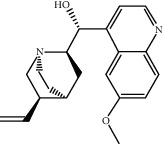	Du Zhong	94175
MOL000296	Hederagenin	36.91	0.75	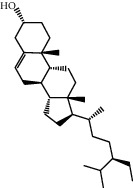	Fu Ling (*Poria Cocos*)	NA
MOL000273	16alpha-Hydroxydehydrotrametenolic acid	30.93	0.81	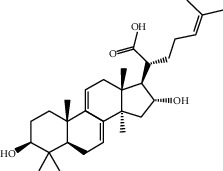	Fu Ling	10743008
MOL004328	Naringenin	59.29	0.21	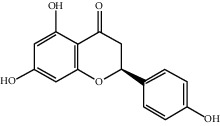	Gan Cao (*Radix Glycyrrhizae*)	439246
MOL000354	Isorhamnetin	49.60	0.31	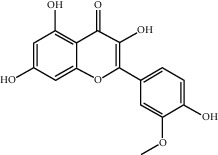	Gan Cao	5281654
MOL000392	Formononetin	69.67	0.21	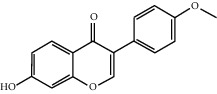	Gan Cao	5280378
MOL002714	Baicalein	33.52	0.21	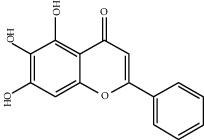	Niu Xi (*Radix Achyranthis Bidentatae*)	5281605
MOL000785	Palmatine	64.60	0.65	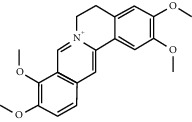	Niu Xi	19009
MOL005344	Ginsenoside rh2	36.32	0.56	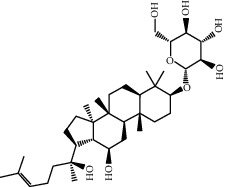	Ren Shen (*Panax Ginseng*)	119307
MOL000787	Fumarine	59.26	0.83	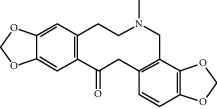	Ren Shen	4970
MOL001558	Sesamin	56.55	0.83	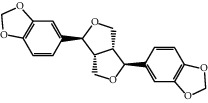	Xi Xin (*Herba Asari*)	72307
MOL002962	3-O-Methylviolanone	48.23	0.33	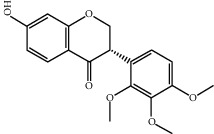	Xi Xin	10019512
MOL011753	5-O-Methylvisamminol	37.99	0.25	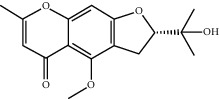	Fang Feng (*Radix Saposhnikoviae*)	441970
MOL000011	Cleomiscosin A	68.83	0.66	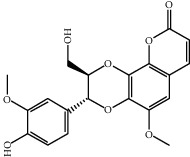	Fang Feng	442510
MOL000449	Stigmasterol	43.83	0.76	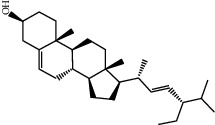	Dang Gui (*Radix Angelicae Sinensis*), Niu Xi, Ren Shen, Shu Di Huang	5280794
MOL000359	3-Epi-beta-Sitosterol	36.91	0.75	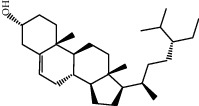	Bai Shao, Chuan Xiong, Fang Feng, Gan Cao, Qin Jiao, Sang Ji Sheng, Shu Di Huang (*Radix Rehmanniae Preparata*)	12303645
MOL000098	Quercetin	46.43	0.28	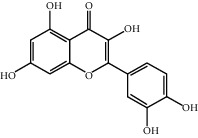	Du Zhong, Gan Cao, Sang Ji Sheng (*Herba Taxilli*), Niu Xi	5280343
MOL000358	Beta-sitosterol	36.91	0.75	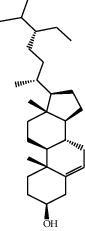	Bai Shao, Dang Gui, Du Huo, Du Zhong, Fang Feng, Niu Xi, Qin Jiao, Ren Shen	222284
MOL000422	Kaempferol	41.88	0.24	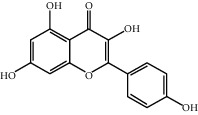	Bai Shao, Du Zhong, Gan Cao, Niu Xi, Ren Shen, Xi Xin	5280863
MOL000173	Wogonin	30.68	0.23	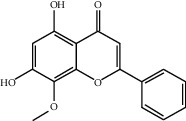	Fang Feng, Niu Xi	5281703
MOL000211	Mairin	55.38	0.78	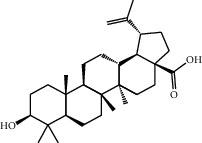	Bai Shao, Du Zhong, Gan Cao	64971
MOL001942	Isoimperatorin	45.46	0.23	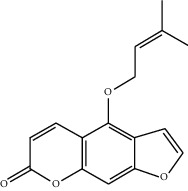	Du Huo, Fang Feng	68081
MOL001941	Ammidin	34.55	0.22	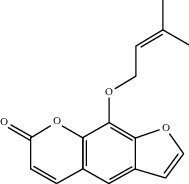	Du Huo, Fang Feng	10212

Abbreviations: DHJSD, Duhuo Jisheng Decoction; OB, oral bioavailability; DL, drug-likeness.

**Table 2:** Basic information of some key targets of DHJSD against osteoporosis.

UniProt ID	Gene symbol	Protein names	Degree
P35354	PTGS2	Prostaglandin G/H synthase 2	144
P03372	ESR1	Estrogen receptor	98
P0DP23	CALM1	Calmodulin-1	96
P07900	HSP90AA1	Heat shock protein HSP 90-alpha	96
P10275	AR	Androgen receptor	85
P35228	NOS2	Nitric oxide synthase, inducible	80
P23219	PTGS1	Prostaglandin G/H synthase 1	74
Q15596	NCOA2	Nuclear receptor coactivator 2	70
P24941	CDK2	Cyclin-dependent kinase 2	68
P37231	PPARG	Peroxisome proliferator-activated receptor gamma	68
P49841	GSK3B	Glycogen synthase kinase-3 beta	65
Q92731	ESR2	Estrogen receptor beta	62
P20248	CCNA2	Cyclin-A2	59
P27487	DPP4	Dipeptidyl peptidase 4	57
P07550	ADRB2	Beta-2 adrenergic receptor	52
P19793	RXRA	Retinoic acid receptor RXR-alpha	52
Q16539	MAPK14	Mitogen-activated protein kinase 14	51
O14757	CHEK1	Serine/threonine-protein kinase Chk1	48
P22303	ACHE	Acetylcholinesterase	34
P08709	F7	Coagulation factor VII	33
Q15788	NCOA1	Nuclear receptor coactivator 1	25
P35968	KDR	Vascular endothelial growth factor receptor 2	24
P25100	ADRA1D	Alpha-1D adrenergic receptor	15
P06401	PGR	Progesterone receptor	15
P31645	SLC6A4	Sodium-dependent serotonin transporter	15
P35372	OPRM1	Mu-type opioid receptor	14
P21728	DRD1	D (1A) dopamine receptor	12
P08235	NR3C2	Mineralocorticoid receptor	12
Q01959	SLC6A3	Sodium-dependent dopamine transporter	12
P27338	MAOB	Amine oxidase [flavin-containing] B	10
Q92934	BCL2	Bcl2-associated agonist of cell death	8
P00918	CA2	Carbonic anhydrase 2	8
P42574	CASP3	Caspase-3	8
Q04206	RELA	Transcription factor p65	7
P31749	AKT1	RAC-alpha serine/threonine-protein kinase	6
Q07812	BAX	Apoptosis regulator BAX	6
P05412	JUN	Transcription factor AP-1	6
O95150	TNFSF15	Tumor necrosis factor ligand superfamily member 15	5
P55211	CASP9	Caspase-9	4
P24385	CCND1	G1/S-specific cyclin-D1	4
P03956	MMP1	Interstitial collagenase	4
P08588	ADRB1	Beta-1 adrenergic receptor	3
P35869	AHR	Aryl hydrocarbon receptor	3
P15121	AKR1B1	Aldo-keto reductase family 1 member B1	3
Q14790	CASP8	Caspase-8	3
P06493	CDK1	Cyclin-dependent kinase 1	3
P05177	CYP1A2	Cytochrome P450 1A2	3
P08684	CYP3A4	Cytochrome P450 3A4	3
P09211	GSTP1	Glutathione S-transferase P	3
Q92819	HAS2	Hyaluronan synthase 2	3
P09601	HMOX1	Heme oxygenase 1	3
P05231	IL6	Interleukin-6	3
P28482	MAPK1	Mitogen-activated protein kinase 1	3
P04150	NR3C1	Glucocorticoid receptor	3
Q03181	PPARD	Peroxisome proliferator-activated receptor delta	3
P14672	SLC2A4	Solute carrier family 2, facilitated glucose transporter member 4	3
P00441	SOD1	Superoxide dismutase [Cu-Zn]	3
Q9H3D4	TP63	Tumor protein 63	3
P15692	VEGFA	Vascular endothelial growth factor A	3
Q13085	ACACA	Acetyl-CoA carboxylase 1	2
P00533	EGFR	Epidermal growth factor receptor	2
P01106	MYC	Myc proto-oncogene protein	2
P02768	ALB	Albumin	1
P01133	EGF	Pro-epidermal growth factor	1
P27361	MAPK3	Mitogen-activated protein kinase 3	1

**Table 3:** The top 10 Gene Ontology (GO) enrichment items.

ID	Description	*P* value	Adjust *P* value	Gene number	GO items
GO:0062197	Cellular response to chemical stress	2.20332*E*− 36	9.75*E − *33	46	Biological process
GO:0010038	Response to metal ion	1.04408 *E*− 35	2.31*E − *32	46	Biological process
GO:0046677	Response to antibiotic	5.44269*E*− 34	8.03*E − *31	43	Biological process
GO:0032496	Response to lipopolysaccharide	8.0729*E*− 34	8.93*E − *31	43	Biological process
GO:0097305	Response to alcohol	1.19658*E*− 33	1.06*E − *30	38	Biological process
GO:0048545	Response to steroid hormone	2.14139*E − *33	1.58*E − *30	45	Biological process
GO:0002237	Response to molecule of bacterial origin	4.25534*E − *33	2.69*E − *30	43	Biological process
GO:0006979	Response to oxidative stress	1.67651*E − *32	9.27*E − *30	47	Biological process
GO:0000302	Response to reactive oxygen species	2.14509*E − *32	1.05*E − *29	37	Biological process
GO:0031667	Response to nutrient levels	1.65363*E − *30	7.32*E − *28	47	Biological process
GO:0004879	Nuclear receptor activity	1.2238*E − *16	3.33*E − *14	14	Molecular function
GO:0098531	Ligand-activated transcription factor activity	1.2238*E − *16	3.33*E − *14	14	Molecular function
GO:0003707	Steroid hormone receptor activity	1.51056*E − *12	2.74*E − *10	12	Molecular function
GO:0061629	RNA polymerase II-specific DNA-binding transcription factor binding	1.0407*E − *11	1.42*E − *09	21	Molecular function
GO:0140297	DNA-binding transcription factor binding	2.02886*E − *11	2.21*E − *09	23	Molecular function
GO:0001228	DNA-binding transcription activator activity, RNA polymerase II-specific	5.32777*E − *11	4.35*E − *09	25	Molecular function
GO:0001216	DNA-binding transcription activator activity	5.59254*E − *11	4.35*E − *09	25	Molecular function
GO:0019902	Phosphatase binding	4.84351*E − *10	3.29*E − *08	16	Molecular function
GO:0020037	Heme binding	5.8588*E − *09	3.54*E − *07	13	Molecular function
GO:0050661	NADP binding	7.53723*E − *09	4.10*E − *07	9	Molecular function
GO:0045121	Membrane raft	3.10297*E − *15	5.63*E − *13	25	Cellular component
GO:0098857	Membrane microdomain	3.33964*E − *15	5.63*E − *13	25	Cellular component
GO:0098589	Membrane region	7.90284*E − *15	8.88*E − *13	25	Cellular component
GO:0005667	Transcription regulator complex	4.33831*E − *10	3.66*E − *08	22	Cellular component
GO:0031983	Vesicle lumen	1.59056*E − *09	1.07*E − *07	19	Cellular component
GO:0090575	RNA polymerase II transcription regulator complex	2.17645*E − *09	1.22*E − *07	14	Cellular component
GO:0000307	Cyclin-dependent protein kinase holoenzyme complex	1.03807*E − *08	5.00*E − *07	8	Cellular component
GO:1902554	Serine/threonine protein kinase complex	2.64297*E − *08	1.11*E − *06	10	Cellular component
GO:0005741	Mitochondrial outer membrane	4.65534*E − *08	1.74*E − *06	13	Cellular component
GO:0060205	Cytoplasmic vesicle lumen	5.57446*E − *08	1.88*E − *06	17	Cellular component

**Table 4:** The enriched 50 possible related pathways of osteoporosis.

ID	Description	*P* value	Adjust *P* value	Gene number
hsa04933	AG*E-*RAGE signaling pathway in diabetic complications	3.2722*E − *31	8.83495*E − *29	34
hsa05418	Fluid shear stress and atherosclerosis	8.02758*E − *26	6.42442*E − *24	34
hsa04657	IL-17 signaling pathway	2.23263*E − *21	6.69789*E − *20	26
hsa04668	TNF signaling pathway	1.93321*E − *20	5.21968*E − *19	27
hsa04659	Th17 cell differentiation	1.77974*E − *17	3.00331*E − *16	24
hsa04210	Apoptosis	5.07009*E − *17	8.0525*E − *16	26
hsa04218	Cellular senescence	1.70327*E − *15	1.9995*E − *14	26
hsa04066	HIF-1 signaling pathway	4.55414*E − *15	4.72929*E − *14	22
hsa01521	EGFR tyrosine kinase inhibitor resistance	1.19998*E − *14	1.11722*E − *13	19
hsa04115	p53 signaling pathway	3.86218*E − *14	3.36383*E − *13	18
hsa05235	PD-L1 expression and PD-1 checkpoint pathway in cancer	1.25365*E − *13	9.95542*E − *13	19
hsa04380	Osteoclast differentiation	1.52063*E − *13	1.14047*E − *12	22
hsa04620	Toll-like receptor signaling pathway	2.26415*E − *13	1.5283*E − *12	20
hsa04660	T Cell receptor signaling pathway	2.26415*E − *13	1.5283*E − *12	20
hsa04012	ErbB signaling pathway	6.6254*E − *13	4.25919*E − *12	18
hsa04151	PI3K-akt signaling pathway	1.24292*E − *12	7.80436*E − *12	34
hsa04370	VEGF signaling pathway	3.47037*E − *12	1.99361*E − *11	15
hsa04915	Estrogen signaling pathway	6.54943*E − *12	3.60403*E − *11	21
hsa04010	MAPK signaling pathway	6.67413*E − *12	3.60403*E − *11	30
hsa04658	Th1 and Th2 cell differentiation	3.05*E − *11	1.55*E − *10	17
hsa04068	FoxO signaling pathway	1.6511*E − *10	7.96064*E − *10	19
hsa04064	NF-kappa B signaling pathway	2.32207*E − *10	1.09993*E − *09	17
hsa04621	NOD-like receptor signaling pathway	1.2042*E − *09	5.16086*E − *09	21
hsa04931	Insulin resistance	3.58386*E − *09	1.423*E − *08	16
hsa04662	B Cell receptor signaling pathway	5.33879*E − *09	2.08909*E − *08	14
hsa04024	cAMP signaling pathway	5.66891*E − *09	2.1641*E − *08	22
hsa04910	Insulin signaling pathway	1.80579*E − *08	6.58868*E − *08	17
hsa04912	GnRH signaling pathway	2.87106*E − *08	1.03358*E − *07	14
hsa04071	Sphingolipid signaling pathway	1.04592*E − *07	3.62048*E − *07	15
hsa04014	Ras signaling pathway	4.64353*E − *07	1.475*E − *06	20
hsa04920	Adipocytokine signaling pathway	5.07934*E − *07	1.57635*E − *06	11
hsa04062	Chemokine signaling pathway	5.25301*E − *07	1.61172*E − *06	18
hsa04110	Cell cycle	1.11163*E − *06	3.15936*E − *06	14
hsa04630	JAK-STAT signaling pathway	1.15731*E − *06	3.25493*E − *06	16
hsa00140	Steroid hormone biosynthesis	1.30408*E − *06	3.59286*E − *06	10
hsa04930	Type II diabetes mellitus	9.82492*E − *06	2.50258*E − *05	8
hsa05323	Rheumatoid arthritis	1.03843*E − *05	2.62033*E − *05	11
hsa04928	Parathyroid hormone synthesis, secretion and action	3.6295*E − *05	8.67227*E − *05	11
hsa03320	PPAR signaling pathway	6.67976*E − *05	0.000155477	9
hsa04020	Calcium signaling pathway	7.36869*E − *05	0.000168606	15
hsa04310	Wnt signaling pathway	9.54755*E − *05	0.000213045	13
hsa04929	GnRH secretion	0.000116074	0.000254797	8
hsa04022	cGMP-PKG signaling pathway	0.000147423	0.000321001	13
hsa04015	Rap1 signaling pathway	0.00041579	0.000863564	14
hsa04666	Fc gamma R-mediated phagocytosis	0.00195976	0.003834313	8
hsa04152	AMPK signaling pathway	0.002019554	0.003922874	9
hsa01212	Fatty acid metabolism	0.002098286	0.003989699	6
hsa04923	Regulation of lipolysis in adipocytes	0.002098286	0.003989699	6
hsa04150	mTOR signaling pathway	0.010773083	0.018409699	9
hsa04918	Thyroid hormone synthesis	0.031361971	0.049810189	5

## Data Availability

The data used to support the findings of this study are included within the article.
